# Supplementary material for: Investigation of the Effect of Particle Surface Charge and Dispersion Stability on Latex Behavior in Cement Using Non-Ionic and Traditional Latexes
Source: Materials (Basel). 2022 Sep 5;15(17):6145. doi: 10.3390/ma15176145 (PMC9458191; doi:10.3390/ma15176145)
Supplement: Supplementary file 1 [file materials-15-06145-s001.zip › materials-1823682-supplementary.pdf]

Text S1: Preparation of Styrene-Butyl Acrylate (St-BA) latex. Styrene and butyl acrylate (100 g total, *w*/*w* = 2/3) and sodium dodecyl benzene sulfonate (0.50 g) were poured into a beaker at room temperature. The mixture was then dispersed in 500 mL of distilled water at 600 rpm for 30 min. Upon full dispersion, 0.85 g of potassium persulfate was dissolved in the monomer dispersion; then, the dispersion was heated to 55 °C, purged with N_2 and 50 mL of solution containing 0.11 g of sodium hydrogen sulfite was added to by a peristaltic pump at a rate of 0.33 mL/min, after addition, the system was kept at 55 °C for another 0.5 h. Finally, unreacted monomers were removed by vacuum (3–5kPa at 30 °C for 2 h), and the resultant latex was stored for further use.
